# ﻿A new species and new records of *Solanum* (Solanaceae) from Colombia

**DOI:** 10.3897/phytokeys.195.78832

**Published:** 2022-05-09

**Authors:** Juan David Tovar, Leandro Lacerda Giacomin

**Affiliations:** 1 Programa de Pós-Graduação em Botânica, Instituto Nacional de Pesquisas da Amazônia, Av. André Araújo, 2936, Manaus, AM, 69060-001, Brazil Instituto Nacional de Pesquisas da Amazônia Manaus Brazil; 2 Programa de Pós-Graduação em Biodiversidade, Universidade Federal do Oeste do Pará, Rua Vera Paz, sn, Santarém, PA, 68040-255, Brazil Universidade Federal do Oeste do Pará Santarém Brazil; 3 Departamento de Sistemática e Ecologia, Centro de Ciências Exatas e da Natureza, Universidade Federal da Paraíba, Cidade Universitária, João Pessoa, PB, 58051-090, Brazil Universidade Federal da Paraíba João Pessoa Brazil

**Keywords:** distribution, nightshades, *Solanum* clade Brevantherum, *Solanum* section *Geminata*, tropical Andes, Andes tropicales, distribución, *Solanum* clado Brevantherum, *Solanum* sección *Geminata*

## Abstract

We describe a new species of the Geminata clade of *Solanum* from Colombia and provide new distributional records for two additional *Solanum* species, recorded here for the first time in Colombia. *Solanumbohsii* J.D. Tovar, **sp. nov.** is morphologically similar to *S.chlamydogynum* Bitter from Venezuela of the *Solanumsessile* species group (Geminata clade). These two species can be distinguished by trichome morphology, as well as colour and density of the indumentum. In addition, we report new range expansions into Colombia for two species: *S.tanysepalum* S.Knapp (Geminata clade) known previously only from Venezuela and *S.verecundum* M.Nee (Brevantherum clade) from Ecuador and Peru.

## Abstract (es)

Describimos una nueva especie del clado Geminata de *Solanum* de Colombia y proveemos nuevos datos de distribución para otras dos especies de *Solanum*, registradas aqui por primera vez en Colombia. *Solanumbohsii* J.D. Tovar, **sp. nov.** es morfológicamente similar con *S.chlamydogynum* Bitter de Venezuela del grupo de especies de *Solanumsessile* (Geminata clade). Estas dos especies se diferencian por el tipo de tricomas, color y densidad de su indumento. Además, reportamos nuevos registros de dos especies que expanden sus rangos de distribucion a Colombia: *S.tanysepalum* S. Knapp (clado Geminata) conocida previamente solo de Venezuela y *S.verecundum* M. Nee (clado Brevantherum) de Ecuador y Perú.

## ﻿Introduction

*Solanum* L., with ca. 1,250 species, is the largest genus in the Solanaceae and one of the 10 most species-rich genera of flowering plants (Frodin 2004; Gagnon et al. 2022). The genus has a worldwide distribution with a centre of diversity in South America concentrated in the Andes (Knapp 2002a). A total of 164 species, with 10 of them endemic, are found in Colombia alone, where *Solanum* species occur in all biogeographic regions of the country, from sea level to 4,200 m in elevation (Orozco et al. 2015).

Molecular studies have divided *Solanum* into 12 major clades (Bohs 2005; Weese and Bohs 2007; Särkinen et al. 2013; Gagnon et al. 2022) that have redefined the infrageneric classification of the genus and are being used as informal infrageneric groups in *Solanum*. The Brevantherum and Geminata clades are among the largest of the non-spiny neotropical clades of *Solanum* with ca. 100 and 150 species, respectively (Knapp 2008; Giacomin 2015). The two clades have similar distributions with centres of diversity and endemism in the Atlantic Forest of Brazil and the tropical Andes (see Knapp et al. 2015; Giacomin 2015). Both clades are unarmed and woody (shrubs or trees) but differ in their trichome type and inflorescence position and morphology. Species of the Brevantherum clade have mostly stout and highly branched inflorescences positioned in branch forks (i.e. terminal inflorescences in dichasially branching stems) and most species have stellate trichomes (Giacomin 2015). Members of the Geminata clade, in contrast, all lack stellate trichomes and have mostly monochasial branching with inflorescences that are leaf-opposed or rarely terminal or internodal (Knapp 2008; Knapp et al. 2015).

Here we describe a new species for the Geminata clade and provide new records of two species from the Brevatherum and Geminata clades of *Solanum* for Colombia with field photos. These discoveries were made during herbarium and fieldwork conducted for a broader phylogenetic study of the Geminata clade. The discovery of the new species from the Geminata clade is not surprising considering members of this clade have an intricate taxonomy due to similar morphology (Knapp 2008) and are often poorly known because they occur in forest understorey habitats, where they are often inconspicuous, locally rare and rarely collected (Knapp et al. 2015). In fact, most undetermined specimens of *Solanum* in tropical herbaria are from the Geminata clade and it is, hence, not surprising that a new species was found during our study of the clade in Colombia.

## ﻿Methods

We examined specimens from the FAUC, HUA, HUQ and JBB Herbaria (acronyms from Index Herbariorum; http://sweetgum.nybg.org/science/ih). For the new species here described, duplicates of paratypes are still awaiting distribution to other countries. Descriptions are based on field observations and herbarium specimens. Preliminary conservation status assessments were done using the IUCN Red List Categories and Criteria (IUCN 2017), based on extent of occurrence (EOO) and area of occupancy (AOO) calculated with the GeoCat tool (www.geocat.kew.org; Bachman et al. 2011). For the AOO calculation, a cell size of 2 km^2^ was used. The morphological cluster species concept of Mallet (1995) was used in defining a species.

## ﻿Taxonomic treatment

### ﻿ Solanum bohsii

Taxon classificationPlantaeSolanalesSolanaceae

J.D. Tovar sp. nov.

DC9D91D9-C9CD-594E-B38C-5E515BECD3BE

urn:lsid:ipni.org:names:77297478-1

Figs 1 and 2

#### Diagnosis.

Like *Solanumchlamydogynum* Bitter, but with a translucent indumentum of unbranched or, at most, furcate trichomes restricted to the veins of the leaves (vs. dendritic, more abundant and ochraceous in *S.chlamydogynum*), cucullate calyx lobes (vs. non-cucullate in *S.chlamydogynum*) and glabrous ovaries (vs. densely pubescent in *S.chlamydogynum*).

#### Type.

Colombia. Risaralda: Municipio de Pereira, Parque Regional Natural Ucumari, sector el cedral, 4°42'16"N, 75°32'20"W, 2100 m elev., 15 Nov 2020 (fl, fr), *J.D. Tovar & A.F. Bohorquez 484* (holotype: FAUC [FAUC36396]; isotypes: COL, FMB, HUA).

#### Description.

Shrubs or small trees, 2–7 m tall; stems winged, greenish-brown when young, turning brown with age, young stems pubescent with translucent simple or furcate 4–8-cellular trichomes. Sympodial units difoliate and geminate, leaves ovate to obovate, glabrous adaxially or with translucent trichomes along the mid-rib and secondary veins like those on stem, abaxially pubescent with trichomes in the mid-rib and along the secondary and tertiary veins; major leaves 18–35 (45) × 12–16 cm, with 12–15 pairs of main lateral veins, these often strongly parallel, the apex acute, base oblique and decurrent on to petiole; petioles 1–2 cm long, pubescent with translucent trichomes, like those on stem; minor leaves differing only in size, not in shape, 8–9.5 × 4–5 cm, with 6–9 pairs of main lateral veins, adaxially and abaxially pubescent along the mid-rib and main lateral veins like those on stem, the apex acute or rounded, base oblique and decurrent on to petiole; petiole 0.5.-0.7 cm long, pubescent with translucent trichomes, like those on stem. Inflorescences leaf-opposed, forked and erect, 20–50 flowered, peduncles 1.5–2.5 cm, with unbranched and uniseriate trichomes, like those on stem, pedicel scars densely spaced, not overlapping, pedicels 0.5–1 cm long, deflexed, thickened at the apex and purple in live plants. Buds globose, with the corolla strongly exserted from the calyx tube prior to anthesis. Flowers 5–6-merous, all perfect; calyx tube cyathiform, 2–3 mm long, the lobes deltoid abruptly reduced and hooded at the apex, 1.5–2 × 1.8–2.2 mm, abaxially glabrous or densely pubescent with trichomes like those of the young stem and with a tuft of hairs at apex; corolla 1.5–2 cm in diameter, white, fleshy, lobed ca. ¾ of the way to the base, the lobes 0.7–0.9 × 3–4 mm spreading or deflexed at anthesis, glabrous and cucullate at the tips; anthers 4–6 × 1.2–1.7 mm, poricidal at the tips, sagittate at the base; free portion of the filaments 0.2–1.2 mm long; ovary glabrous; style 0.5–0.7 mm long, terete, glabrous, stigma capitate, light green in live plants. Fruit a globose green berry, 1–1.2 cm in diameter, glabrescent or with a few scattered trichomes like those on stem and an apical scar, green at maturity; fruiting pedicels 1.5–2 cm long, erect, woody and somewhat rugose, distally enlarged, the calyx constricted and with lobes woody in fruit. Seeds ca. 80 per fruit, 2.5–3.5 × 2–2.5 mm, flattened-reniform, greyish when dry, the margins incrassate, the surfaces minutely pitted. Chromosome number not known.

**Figure 1.**
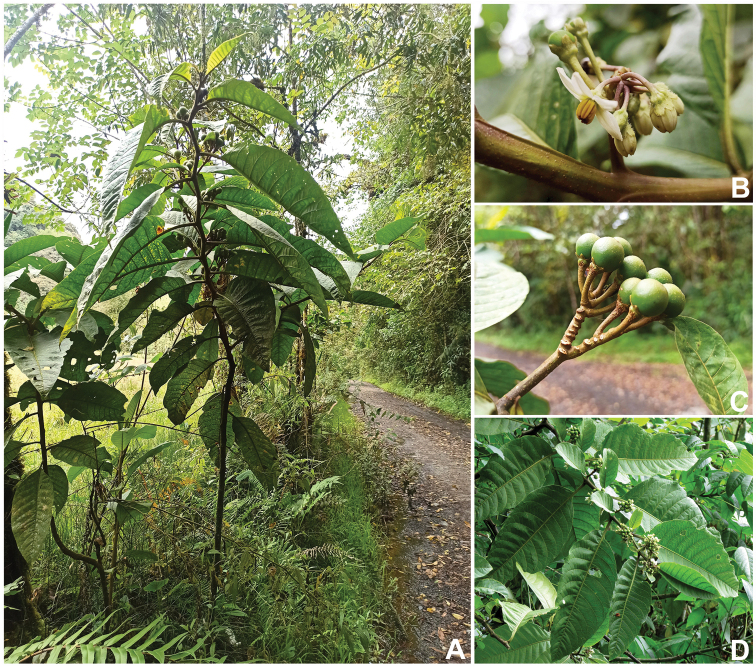
*Solanumbohsii* J.D. Tovar **A** plant habit **B** flowers and buds **C** infructescence **D** sympodial units.

**Figure 2.**
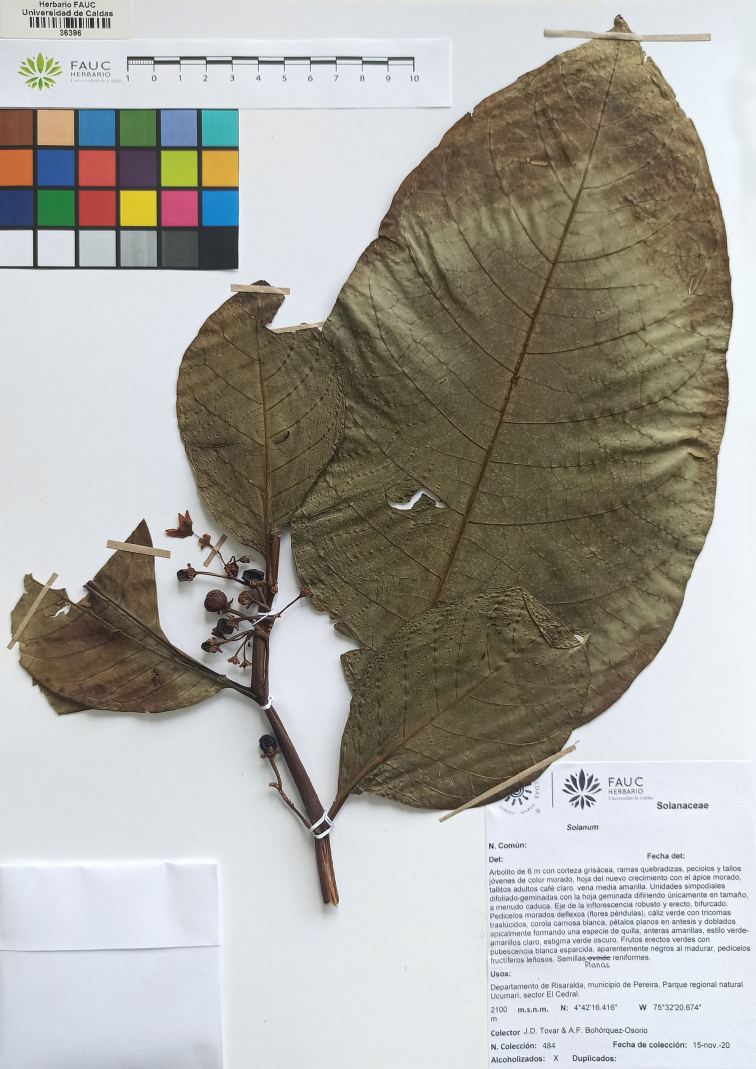
Holotype of *Solanumbohsii* J.D. Tovar [*J.D. Tovar & A.F. Bohorquez 484* (FAUC 36396)].

#### Distribution and ecology.

**Figure 3.**
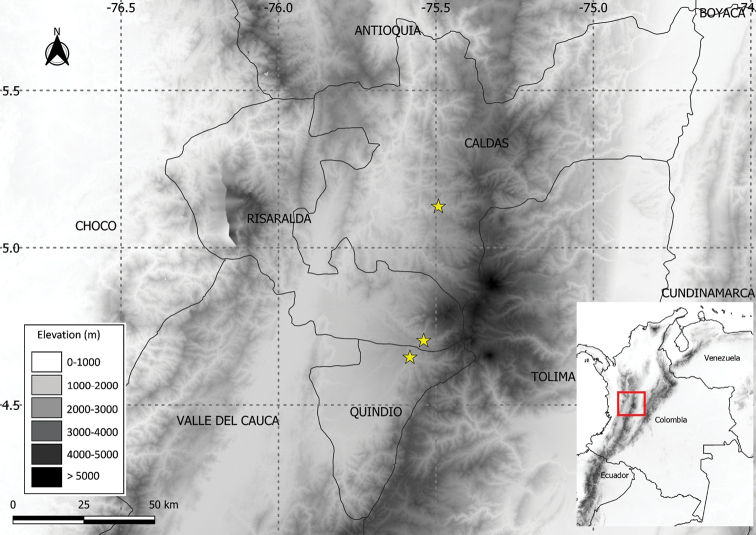
Distribution of *Solanumbohsii* J.D. Tovar (yellow stars).

*Solanumbohsii* is known only from three localities in the western slopes of the central Andean cordillera in Colombia in the Departments of Caldas, Quindio and Risaralda (Fig. 3) where it inhabits secondary forest edges between 1,900–2,300 m elevation, forming groups of up to 10 individuals.

#### Phenology.

According to the collections studied, *S.bohsii* produces flowers and fruits throughout the year.

#### Etymology.

The specific epithet honours Lynn Bohs, an American botanist and expert in the Solanaceae family, who has made great contributions to the understanding of systematics and evolution of the genus *Solanum* over the last 30 years.

#### Preliminary conservation status.

Endangered [EOO (100 km^2^) and AOO (12 km^2^)]. We assign *S.bohsii* a preliminary IUCN Red List status of endangered (EN), based on assessment criteria B2 a, b (i,ii,iv) (IUCN 2017).

#### Notes.

According to its morphology, *S.bohsii* is a member of the *Solanumsessile* species group (sensu Knapp 1991; 2002b) with difoliate and geminate sympodial units, large leaves and large branched inflorescences and ovoid-reniform seeds. It is easily distinguished from the other species in the group by its large leaves up to 45 cm long and the secondary veins strongly parallel in mature leaves. *Solanumbohsii* is morphologically similar to *S.chlamydogynum* from Venezuela with which it shares the winged stems, the fleshy large flowers with cucullate lobes and the presence of trichomes in both leaf surfaces. However, trichomes of *S.bohsii* are simple or, at most, furcate, translucent and less abundant compared to *S.chlamydogynum* which has dendritic ochraceous trichomes. Furthermore, inflorescence peduncles are less pubescent in *S.bohsii*, while densely pubescent with ochraceous trichomes in *S.chlamydogynum*.

*Solanumbohsii* has distinctive mature fruits in dry material with a network-like pericarp, resembling the venation of leaves. From the other species in the *Solanumsessile* species group, it is easily recognised either by the pubescence, the winged stems, the large leaves or the large flowers with cucullate lobes (see Knapp 1991; 2002b).

#### Paratypes.

**Colombia. Caldas**: Municipio de Neira, antigua fábrica de cementos Caldas, 2,026 m elev., 7 Jul 2001 (fl, fr), Camilo Rivera, Andres Castellanos, *C. Arbelaez & W. Acosta 8* (FAUC). **Quindío**: Municipio de Salento, bosque frente al molino, 1,900–2,000 m elev., 9 May 1997 (fl, fr), *W. Vargas 3820* (HUQ). **Risaralda**: Municipio de Pereira, cerca de la entrada del Parque Regional Ucumari, 1,800 m elev., 10 Aug 2014 (fl, fr), *J.D. Tovar 114* (JBB); Municipio de Pereira, Parque Regional Natural Ucumari, sector el cedral, 4°42'16"N, 75°32'20"W, 2,100 m elev., 15 Nov 2020 (fl), *J.D. Tovar & A.F. Bohorquez 481* (FAUC); Municipio de Pereira, Parque Regional Natural Ucumari, sector el cedral, 4°42'16"N, 75°32'20"W, 2,100 m elev., 15 November 2020 (fl), *J.D. Tovar & A.F. Bohorquez 482* (FAUC); Municipio de Pereira, Parque Regional Natural Ucumari, sector el cedral,4°42'16"N, 75°32'20"W, 2,100 m elev., 15 Nov 2020 (fl, fr), *J.D. Tovar & A.F. Bohorquez 483* (FAUC); Municipio de Pereira, Parque Regional Natural Ucumari, sector el cedral,4°42'16"N, 75°32'20"W, 2,100 m elev., 15 Nov 2020 (fl), *J.D. Tovar & A.F. Bohorquez 485* (FAUC); Municipio de Pereira, Parque Regional Natural Ucumari, sector el cedral,4°42'16"N, 75°32'20"W, 2,100 m elev., 15 Nov 2020 (fr), *J.D. Tovar & A.F. Bohorquez 486* (FAUC). Municipio de Pereira, Santuario de flora y fauna Otún Quimbaya, 12 Feb 2016 (fl, fr). *A. Orejuela* et al. *2630* (JBB); Municipio de Pereira, Reserva Natural Ucumari, La Pastora, camino a Ceylan, 5°06'47"N, 75°53'16"W, 2,300 m elev., 30 Jul 2006 (fl, fr), *F.J. Roldan* et al. *4010* (HUA); Municipio de Pereira, Valle del río Otún, Parque Regional Ucumari, El Cedral, 4°02"N, 79°31'W, 1,980–2,000 m elev., 20 Feb 1990 (fl, fr), *T.B. Croat & M.P. Galeano 70797* (HUA, MO).

### ﻿New records for *Solanum* in Colombia

#### ﻿ Solanum tanysepalum

Taxon classificationPlantaeSolanalesSolanaceae

S. Knapp, Brittonia 38: 284. 1986

7C6878FA-B009-5823-98B4-4D937C11D26D

##### Type.

Venezuela. Aragua: Parque Nacional Henri Pittier, Portachuelo to Pico Periquito trail, W of Estación Biológica Rancho Grande, premontane to montane rainforest, 1,100–1,400 m elev., 10°21'N, 67°42'W, 22 Oct 1984, *S. Knapp & J. Mallet 6856* (holotype: MY; isotypes: BH, F! photo, K!, MO! photo, NY! photo, US! photo, VEN).

##### Notes.

**Figure 4.**
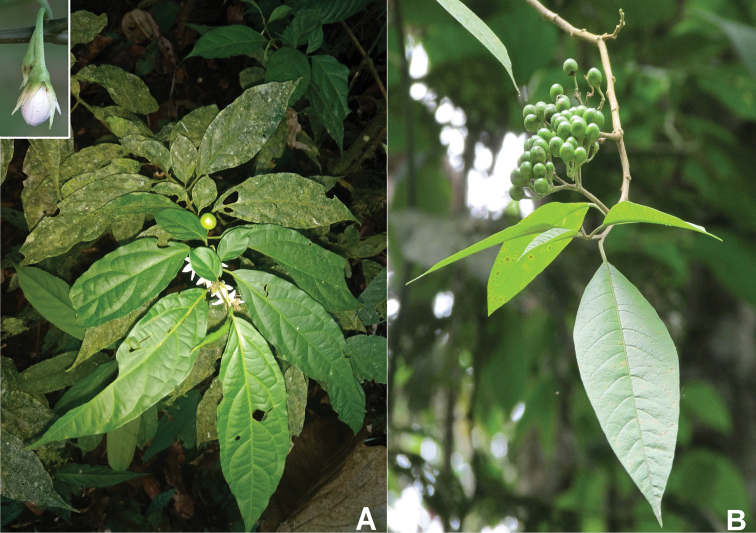
New records of *Solanum* from Colombia **A***Solanumtanysepalum* S.Knapp (upper left corner: detail of calyx lobes; Geminata clade) **B***Solanumverecundum* M.Nee (Brevantherum clade).

**Figure 5.**
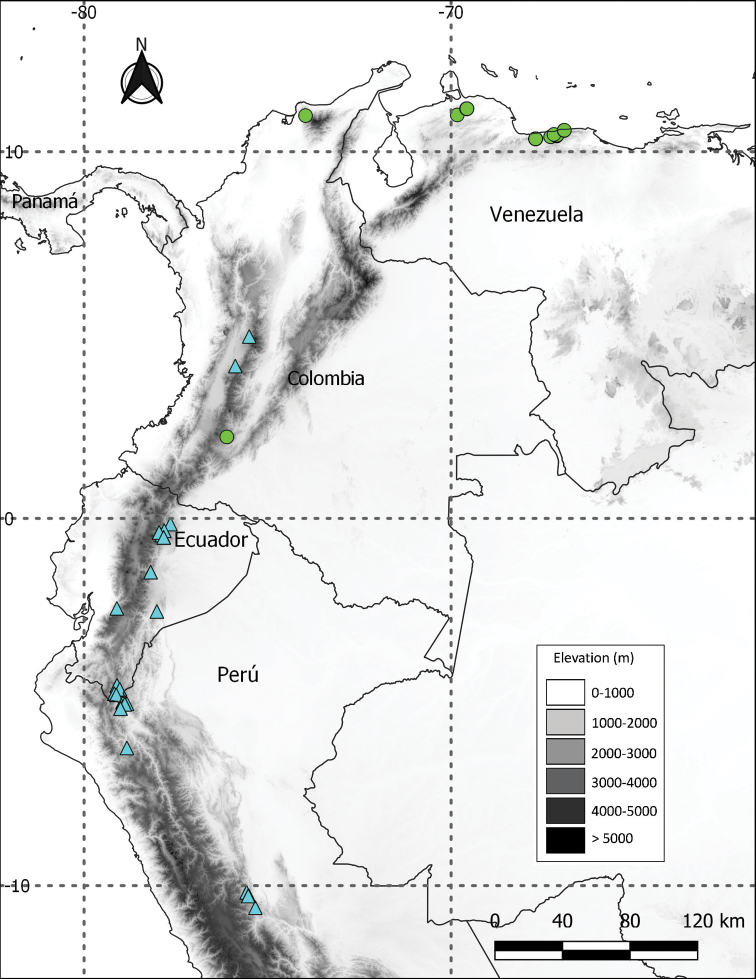
Distribution of *S.tanysepalum* S.Knapp (green circles) and *S.verecundum* M.Nee (cyan triangles).

*Solanumtanysepalum* (Fig. 4a) is a species previously known only from the Cordillera de la Costa in Venezuela in cloud forest from 1,000 to 1,700 m elevation. Our new records presented here extend the distribution to the departments of Huila and Magdalena in Colombia (Fig. 5). The species belongs to the *Solanumarboreum* species group in the Geminata clade and can be easily recognised by the long-acuminate calyx lobes that are persistent and somewhat woody in fruit (Knapp 2002a, b). More information and a complete description are available at: https://solanaceaesource.myspecies.info/solanaceae/solanum-tanysepalum.

##### Specimens examined.

**Colombia. Huila**: Municipio de La Plata, reserva natural Meremberg, bosques contiguos al cementerio Solanaceaer, 2°13'09.6"N, 76°06'39.9"W, 2,300 m elev., 15 May 2019 (fl,fr), *J.D. Tovar 446* (JBB); Municipio de La Plata, reserva natural Meremberg, bosques contiguos al cementerio Solanaceaer, 2°13'09.6"N, 76°06'39.9"W, 2300 m elev., 15 May 2019 (fl, fr), *J.D. Tovar 447* (JBB); Municipio de La Plata, reserva natural Meremberg, bosques contiguos al cementerio Solanaceaer, 2°13'09.6"N, 76°06'39.9"W, 2,300 m elev., 15 May 2019 (fl, fr), *J.D. Tovar 448* (JBB); Municipio de La Plata, reserva natural Meremberg, bosques contiguos al cementerio Solanaceaer, 2°13'09.6"N, 76°06'39.9"W, 2,300 m elev., 15 May 2019 (fl, fr), *J.D. Tovar 449* (JBB). **Magdalena**: Sierra nevada de Santa Marta, in forest N of finca Cecilia, quebrada Indiana, 10°59'N, 73°58'W, 1,820 m elev., 31 Aug 1972 (fr), *J.H. Kirkbride* Jr. 2021 (NY).

#### ﻿ Solanum verecundum

Taxon classificationPlantaeSolanalesSolanaceae

M.Nee, Kurtziana 28: 137, 2000.

E89D71CE-0909-5FDB-B77C-47AF1F2C02B6

##### Type.

Ecuador. Sucumbios: El Salado, colecciones en el sendero a la finca del Sr.

Segundo Pacheco, 1,400 m elev., 13 October 1990, J. Jaramillo, E. Grijalva & M. Grijalva 13285 (holotype: QCA!; isotype: NY! [00381798]).

##### Notes.

*Solanumverecundum* (Fig. 4b) was previously known from montane forests in Peru and Ecuador (Giacomin 2015). Our new records presented here extend the distribution to Colombia to the Departments of Caldas, Quindio and Valle del Cauca (Fig. 5). The species belongs to the Brevantherum clade and is related to the species traditionally included in section Brevantherum Seithe (Tovar et al. 2021). *Solanumverecundum* can be easily recognised by the membranaceous leaves, stellate-lepidote trichomes (with partially fused rays) in both leaf surfaces and puberulous orange-coloured fruits at full maturity. More information and a complete description are available at: https://solanaceaesource.myspecies.info/solanaceae/solanum-verecundum

##### Specimens examined.

**Colombia. Caldas**: Municipio de Villamaria, ruta del condor, carretera entre la Telaraña y La Guyana, 4°57'18.5"N, 75°30’03.3"W, 2,000 m elev., 29 Nov 2021 (fl,fr), *J.D. Tovar & M.A. Buitrago 487* (FAUC). **Quindio**: Municipio de Génova, por la trocha que conduce a Pijao, camino al Cedral, 1,800 m elev., 11 Feb 2016 (fr), *A. Orejuela* et al. *2627* (JBB); Municipio de Génova, vereda La Esmeralda, borde de carretera, 1,750 m elev., 8 Jan 1993 (fr), *C.A. Lopez 61* (HUQ). **Valle del Cauca**: Márgenes del río Bugalagrande, Calamar, 1,680 m elev., 28 Mar 1946 (fr), *J. Cuatrecasas* 20500 (F).

## Supplementary Material

XML Treatment for Solanum bohsii

XML Treatment for Solanum tanysepalum

XML Treatment for Solanum verecundum
